# Predicting Clear Cell Renal Cell Carcinoma Survival Using Kurtosis of Cytoplasm in the Hematoxylin Channel from Histology Slides

**DOI:** 10.1155/2022/7693993

**Published:** 2022-01-13

**Authors:** Jun Wang, Jianhui Chen, Liren Jiang, Qi Wu, Dawei Wang

**Affiliations:** ^1^Department of Urology, Shanghai General Hospital, Shanghai Jiao Tong University School of Medicine, Shanghai 200080, China; ^2^Department of Medical Oncology, Shuguang Hospital, Shanghai University of Traditional Chinese Medicine, Shanghai 200021, China; ^3^Department of Pathology, Shanghai General Hospital, Shanghai Jiao Tong University School of Medicine, Shanghai 200080, China; ^4^Department of Urology, The Sixth Affiliated Hospital of Wenzhou Medical University (The People's Hospital of Lishui), Wenzhou, Zhejiang 323000, China; ^5^Department of Urology, Ruijin Hospital, School of Medicine, Shanghai Jiao Tong University, Shanghai 200025, China

## Abstract

**Purpose:**

Grade-dependent decrease of lipid storage in clear cell renal cell carcinoma (ccRCC) leads to morphology changes in HE sections. This study investigated the role of cytoplasmic features in frozen sections of ccRCC on prognosis using the digital pathology approach.

**Methods:**

We established an automatic pipeline that performed tumor region selection, stain vector normalization, nuclei segmentation, and feature extraction based on the pathologic data from Shanghai General Hospital and The Cancer Genome Atlas database. Extracted features were subjected to survival analysis.

**Results:**

Kurtosis of the cytoplasm in the hematoxylin channel was correlated with progression-free survival (HR 0.10, 95% CI: 0.04–0.24, *p*=6.52^*∗*^10^−7^) and overall survival (HR 0.11, 95% CI: 0.05–0.31, *p*=1.72^*∗*^10^−5^) in ccRCC, which outperformed other texture features in this analysis. Multivariate Cox regression analysis revealed that low kurtosis of cytoplasm in the hematoxylin channel was an independent predictor for a shorter progression-free survival time (*p*=0.044) and overall survival time (*p* = 0.01). Kaplan–Meier survival analysis of progression-free survival and overall survival also showed a significantly worse prognosis in patients with low kurtosis of the cytoplasm in the hematoxylin channel (both *p* < 0.0001). Lower kurtosis of cytoplasm in the hematoxylin channel was associated with higher pathologic grade, less cholesterol ester, and more mitochondrial DNA content.

**Conclusion:**

Kurtosis of the cytoplasm in the hematoxylin channel predicts survival in clear cell renal cell carcinoma.

## 1. Introduction

Clear cell renal cell carcinoma (ccRCC) is histologically characterized by its cholesterol-ester-rich cytoplasmic deposits [1, 2]. Our previous study has shown that abnormal cholesterol metabolism also contributes to ccRCC progression by elevating expression levels of lysosomal acid lipase (LIPA) and aberrant activation of PI3K signaling [3]. However, with increased activation of the lipolysis pathway, high-grade ccRCC actually has less cholesterol ester, rendering cancer cells display eosinophilic cytoplasm [3–5]. Takashi et al. manually divided ccRCC into three categories based on eosinophilic features: clear, mixed, or eosinophilic type. The eosinophilic type displayed a higher proliferative drive and lower differentiation [6]. Thus, in addition to the traditional Fuhrman nuclear and International Society of Urological Pathology (ISUP) grades, features of the cytoplasm also have a role in the survival prediction of ccRCC [7]. The features of cytoplasm images likely contain underutilized biological information and need to be further studied in depth with the support of advanced technology in digital pathology.

We investigated the role of these cytoplasmic features in frozen sections of ccRCC on oncological outcomes. We found that kurtosis of the cytoplasm in the hematoxylin channel predicts the survival of clear cell renal cell carcinoma.

## 2. Materials and Methods

### 2.1. Ethics Statement

Our study was approved by the Institutional Ethics Committee of Shanghai General Hospital. Written informed consents were obtained from all subjects from Shanghai General Hospital. The study design and all testing procedures were performed according to the ethical standards of the Helsinki Declaration II.

### 2.2. Dataset

The investigation was conducted in accordance with ethical standards and was approved by the authors' Institutional Review Board. In this study, a total of 30 ccRCC patients were enrolled from Shanghai General Hospital and treated between November 2013 and November 2015. All of these included patients met the specified inclusion criteria as follows: (i) accepted radical or partial nephrectomy in Shanghai General Hospital and (ii) diagnosed with ccRCC. Cancer tissues were collected from patients who had undergone primary surgical treatment for ccRCC in Shanghai General Hospital and immediately fixed with formalin. A total of 500 images were taken from these patients' HE sections. Tumor, overlapping, necrotic, and fibrotic regions were labeled by two pathologists.

Another 537 ccRCC patients in The Cancer Genome Atlas (TCGA) were also included in this study. Frozen section images were downloaded from the website of TCGA portal on May 18, 2017.

### 2.3. DenseNet Neural Network

A modified DenseNet neural network was created using the framework Keras 2.2.4 [8]. The network received multiscale pathologic image inputs and was trained to identify the tumor region of the slice using the dataset from Shanghai General Hospital. The network structure and training hyperparameters can be found in the data supplement.

### 2.4. Processing Pipeline

**Figure 1:**
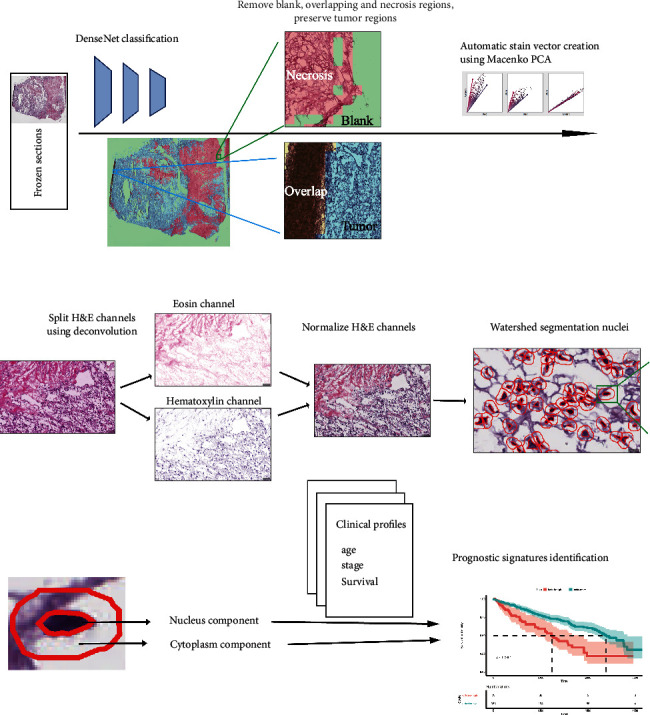
The workflow of histopathology image processing and machine learning analysis in this study. The automatic pipeline includes region selection, stain vector calculation, color deconvolution, intensity normalization, nuclei segmentation, and feature extraction.

The pipeline is shown in Figure 1. Whole slide images (WSIs) of frozen sections of TCGA were divided into patches. All these patches were classified by modified DenseNet. Non-tumor regions were discarded. The tumor regions of the images were subjected to Macenko principal component analysis (PCA), and the stain vectors were applied to the deconvolution process of the images [9]. The hematoxylin channel and eosin channel were normalized across all WSIs. Watershed segmentation was applied to isolate nuclei, and the 4 *μ*m expanded perinucleus region was defined as the cytoplasmic component [10]. The features of nuclear component and cytoplasmic component were extracted.

### 2.5. Feature Extracting

The min, max, mean, median, mean-median difference, std, IQR, MAD, skewness, kurtosis, histogram, energy histogram, and entropy of intensity of the hematoxylin channel and eosin channel were separately extracted using Python 3.6. The median values of these features in each patient were calculated and used for further survival analysis.

### 2.6. ccRCC Grading

The ISUP grading was carried out in accordance with published guidance by an experienced urological pathologist [11].

### 2.7. Mitochondrial DNA Quantification

Mitochondrial DNA content was determined as described previously [12]. In brief, total DNA was isolated using a Tissue DNA Kit (Omega). Quantitative PCR was performed with primers for nuclear and mitochondrial encoded genes. The relative mitochondrial DNA content was determined by the ratio of mitochondrial DNA encoded genes and nuclear encoded genes. Primers for COXII (mitochondrial genome) were CCTGCGACTCCTTGACGTTG and AGCGGTGAAAGTGGTTTGGTT; primers for NQO1 (nuclear genome) were TCATTTCCAGAAAGGACATCACA and CAGAACAGACTCGGCAGGATACT.

### 2.8. Cholesteryl Ester Quantification

Cholesteryl esters in tissue samples were extracted with a 200 *μ*l mixture of chloroform: isopropanol: NP-40 (7 : 11 : 0.1) followed by air drying at 50°C to remove chloroform. Dried lipids were measured using cholesteryl ester total cholesterol and a cholesteryl ester Colorimetric/Fluorometric Assay Kit (BioVision).

### 2.9. Statistical Analysis

Kaplan–Meier survival analysis of progression-free survival (PFS) and overall survival (OS) with hazard ratios (HRs) and 95% confidence intervals (CIs) was stratified by the kurtosis of cytoplasm in the hematoxylin channel. The cut-off value was defined by the method described by Lausen et al. and calculated using the R package survminer (v0.4.9) [13]. Univariate and multivariate Cox regression analyses were conducted to identify the risk score as an independent prognostic factor of PFS for ccRCC patients. R 3.6.1 (http://www.r-project.org/) was used for statistical analysis. A *p* value smaller than 0.05 was regarded as significant.

## 3. Results

### 3.1. Development of the Novel Preprocessing Computational Recognition Model

Based on the general hospital training cohort, a modified DenseNet model was trained to remove nontumor regions of the pathologic image (detailed network structure in the data supplement). In the general hospital test cohort, the accuracy of classification of the tumor region was over 99%. The overall accuracy of the classification of tumor regions on the external subset of the TCGA ccRCC dataset was 95.7%. The representative classification of the images is shown in Figure 1.

### 3.2. The Variation of the Eosin Channel in Frozen Sections Is Large and Requires Normalization

**Figure 2:**
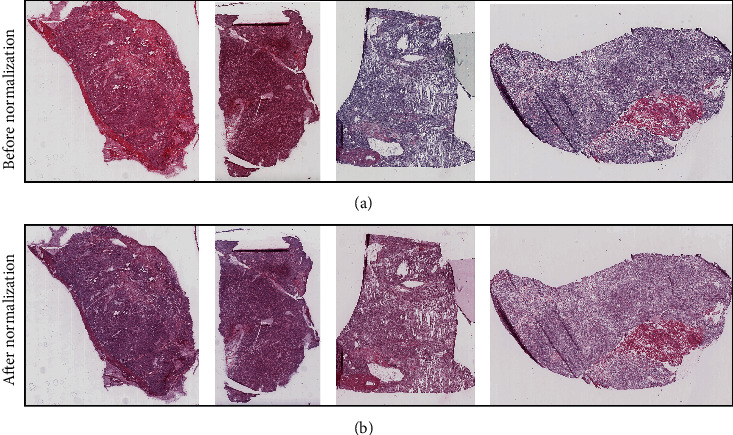
Representative frozen sections before and after color normalization. Histopathology images were subjected to Macenko principal component analysis and applied color normalization. (a) Before color normalization. (b) After color normalization.

We found large variations in eosin intensities in TCGA frozen sections (Figure 2). Thus, we used the deconvolution method to separate the hematoxylin channel and the eosin channel and rescaled both channels to make the samples comparable to each other (Figure 2).

### 3.3. Kurtosis of the Cytoplasm in the Hematoxylin Channel Was Correlated with Prognosis in ccRCC

**Figure 3:**
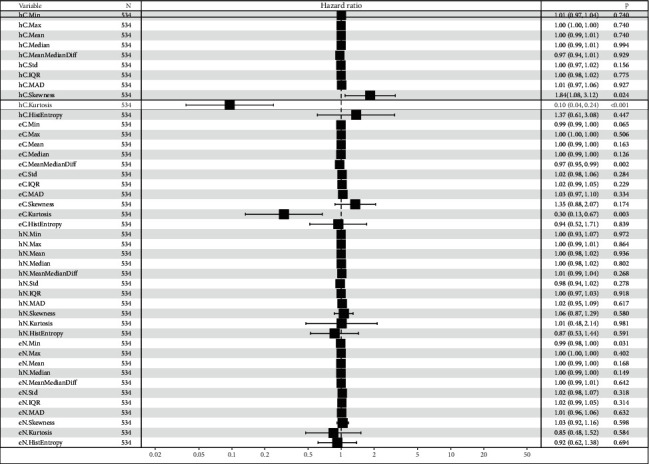
Univariate Cox regression analysis of texture features in both nuclei and cytoplasm. The univariate analysis based on Cox regression was used to assess the correlation between PFS and texture features in both nuclei and cytoplasm. hC: cytoplasm in the hematoxylin channel, eC: cytoplasm in the eosin channel, hN: nuclei in the hematoxylin channel, and eN: nuclei in the eosin channel.

**Figure 4:**
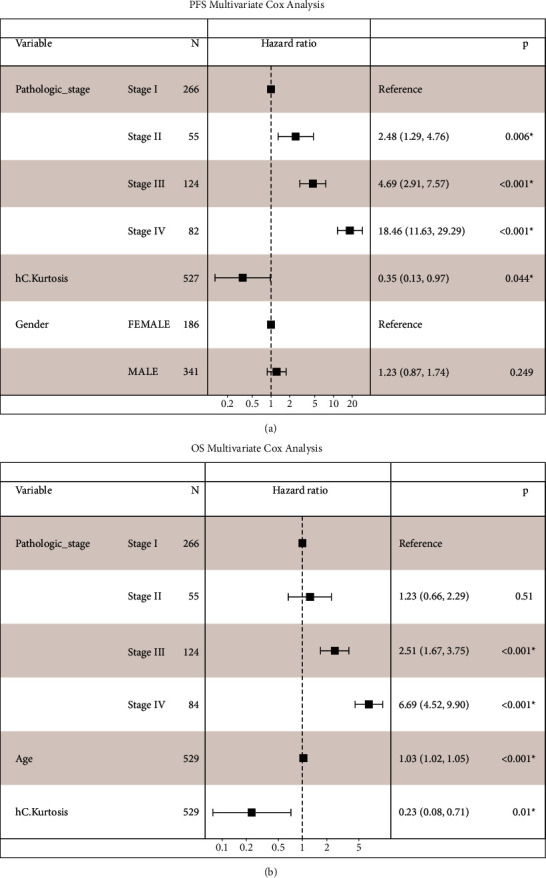
Multivariate Cox regression analysis of progression-free survival and overall survival. Multivariate Cox regression analysis revealed that low kurtosis of cytoplasm in the hematoxylin channel was an independent predictor of a shorter progression-free survival time (*p*=0.044) and overall survival time (*p* = 0.01). hC: cytoplasm in the hematoxylin channel, eC: cytoplasm in the eosin channel, hN: nuclei in the hematoxylin channel, and eN: nuclei in the eosin channel. *∗p* < 0.05.

The texture features, including min, max, mean, median, mean-median, difference, std, IQR, MAD, skewness, kurtosis, histogram energy, and histogram entropy, were separately extracted from hematoxylin channels and eosin channels, respectively, in 537 ccRCC cases with frozen sections and survival data. We next carried out univariate and multivariate Cox regression analyses (Figures 3 and 4). There was a significant difference in PFS between patients with high and low kurtosis of cytoplasm in the hematoxylin channel (HR = 0.095, 95% CI: 0.04–0.24, *p*=6.52^*∗*^10^−7^) (Figure 3) in the univariate Cox regression analysis. The skewness of cytoplasm in the hematoxylin channel, the mean-median difference of cytoplasm in the eosin channel, the kurtosis of cytoplasm in the eosin channel, and the minimum values of nuclei in the eosin channel were also statistically significant variables in the univariate Cox regression analysis. These variables and clinicopathologic factors, including patient age, gender, and tumor stage, were included in the multivariate stepwise Cox regression analysis. Kurtosis of the cytoplasm in the hematoxylin channel (*p*=0.044), tumor stage, and gender entered the final model for PFS (Figure 4(a)). The kurtosis of the cytoplasm in the hematoxylin channel was also correlated with OS (HR = 0.11, 95% CI: 0.05–0.31, *p*=1.72^*∗*^10^−5^) in the univariate Cox regression analysis. Kurtosis of the cytoplasm in the hematoxylin channel (*p*=0.01), tumor stage, and age entered the final multivariate stepwise model for OS (Figure 4(b)). Thus, the kurtosis of the cytoplasm in the hematoxylin channel outperforms all other texture features extracted from frozen sections of ccRCC.

**Figure 5:**
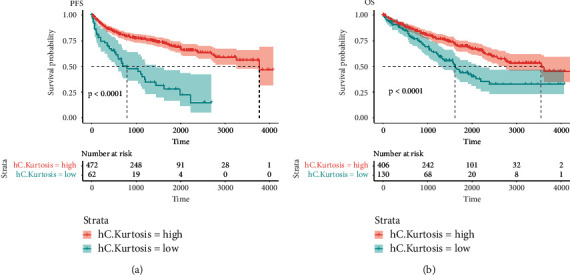
Kaplan–Meier curves of progression-free survival and overall survival stratified by kurtosis. Kaplan–Meier curves of progression-free survival (a) and overall survival (b) stratified by the kurtosis of cytoplasm in the hematoxylin channel. hC: cytoplasm in hematoxylin channel.

The Kaplan–Meier survival analysis of PFS and OS also showed a significantly worse prognosis in patients with low kurtosis of cytoplasm in the hematoxylin channel (both *p* < 0.0001) (Figures 5(a) and 5(b)).

### 3.4. Lower Kurtosis of the Cytoplasm in the Hematoxylin Channel Is Associated with Higher Pathologic Grade

**Figure 6:**
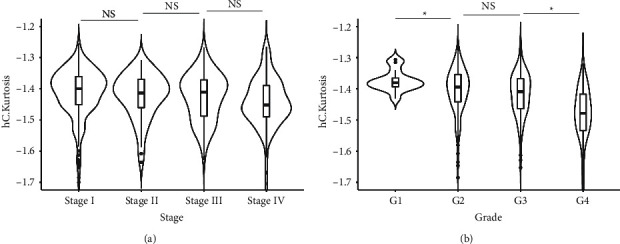
Kurtosis of cytoplasm in the hematoxylin channel in different stages and grades of ccRCC. The boxplots of kurtosis of cytoplasm in the hematoxylin channel in different stages (a) and grades of ccRCC (b) in the TCGA dataset. hC: cytoplasm in hematoxylin channel. NS: not significant.

We compared the kurtosis of cytoplasm in the hematoxylin channel in different pathologic stages and pathologic grades in the TCGA dataset. There was no significant difference between the different pathologic stages (Figure 6(a)). Interestingly, the variation in kurtosis of the cytoplasm in the hematoxylin channel in grade 1 ccRCC seemed smaller than that in other higher grade ccRCC (Figure 6(b)). The kurtosis of cytoplasm in the hematoxylin channel was decreased in higher-grade tumors. The grade 4 ccRCC had a significantly lower kurtosis of the cytoplasm in the hematoxylin channel than the grade 3 ccRCC (Figure 6(b)). However, the difference between grade 2 and grade 3 was not statistically significant.

**Figure 7:**
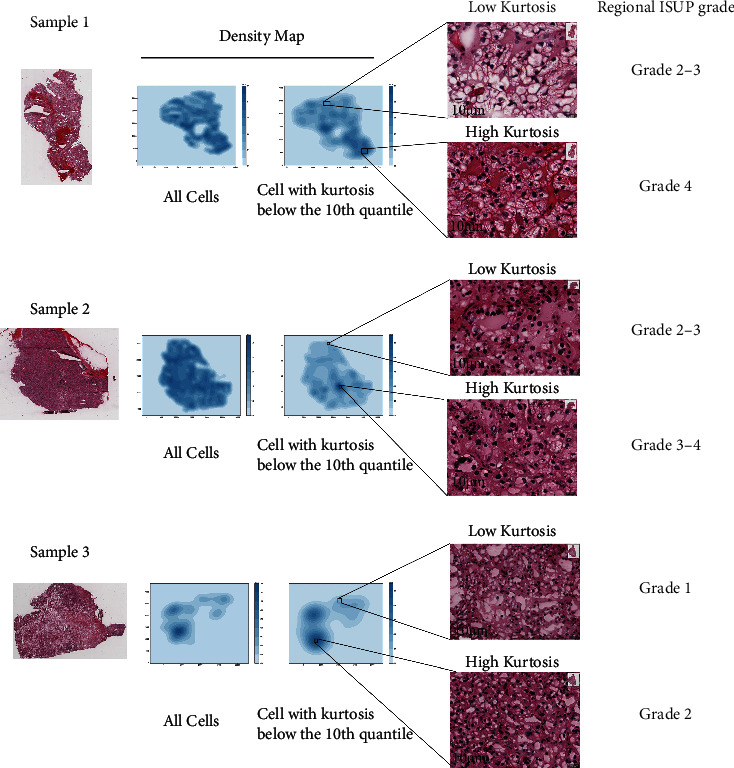
Low-kurtosis areas are associated with a higher grade of ccRCC. Three representative pathological slices with a corresponding density heatmap with all cells and only cells from kurtosis below the 10th quantile.

Due to tumor heterogeneity, the grades of different parts of the tumor are different. We found that kurtosis in the tumor was not evenly distributed. We selected the pathological slices of three patients from the TCGA database for display (Figure 7). We found that low kurtosis areas clustered together, and the regional tumor grades in these areas were higher (Figure 7).

### 3.5. ccRCC Tissue Samples with Lower Kurtosis of Cytoplasm in the Hematoxylin Channel Have Less Cholesterol Ester and More Mitochondrial DNA Content

**Figure 8:**
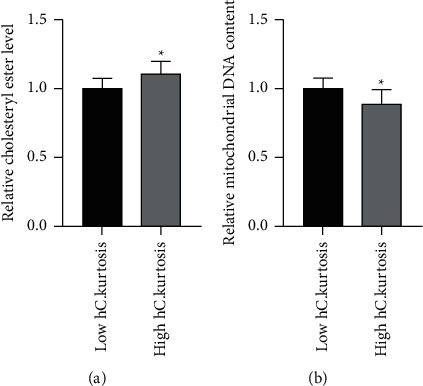
ccRCC tissue samples with lower kurtosis of cytoplasm in the hematoxylin channel have less cholesterol ester and more mitochondrial DNA content. The boxplots of cholesterol ester (a) and mitochondrial DNA content (b) in low versus high kurtosis of cytoplasm in the hematoxylin channel. hC: cytoplasm in hematoxylin channel. *∗p* < 0.05.

To test whether the lower kurtosis of cytoplasm in the hematoxylin channel resulted from decreased cholesterol ester, we measured cholesterol ester in ccRCC tissue samples from Shanghai General Hospital. The low kurtosis group had less cholesterol ester (*p* < 0.05, Figure 8(a)). Furthermore, we compared mitochondrial DNA in ccRCC tissue with low versus high kurtosis of cytoplasm in the hematoxylin channel. The low kurtosis group had higher mitochondrial DNA content (*p* < 0.05, Figure 8(b)).

## 4. Discussion

Currently, partial nephrectomy is the mainstream surgical method for small and medium-sized localized renal tumors [14]. However, renal tumor recurrence occurred in some patients after partial nephrectomy [15, 16]. Local recurrence rates of 1.2% to 9% have been reported [15, 17]. In fact, tumor tissue samples obtained during partial nephrectomy are underutilized. Using rapid intraoperative freezing of pathology to make predictions of pathological types and grades in a very short time may be used to optimize surgical decisions. When the surgeon immediately knows that the patient's tumor is poorly differentiated and highly malignant, and the prognosis is poor, whether the surgeon still preserves the kidney is a question worth studying. Traditional distinguishing renal cell carcinoma by the naked eye of experienced pathologists remains labor intensive and time consuming. Digital pathology and artificial intelligence technology make it possible to carry out this kind of research.

In this study, a deep learning neural network is only used as a tool to remove unrelated regions. We still used simple and interpretable features as prognostic signatures. The kurtosis describes the shape of a probability distribution and is a measure of the relative peakedness of a distribution. We speculate that the reduction of intracellular lipids makes the cells no longer have a clear cell-like cytoplasm, and the cells are filled with more nonlipid parenchyma and organelles, which reduces the sharp edge in the image. This is consistent with the results in Figure 8. The low kurtosis group had less cholesterol ester and more mitochondrial DNA content. ccRCC with higher malignancy seems to bypass HIF1a-driven lipid storage and increase the activation of *β* oxidation [4, 5]. However, its implication is still not clear.

It is interesting that meaningful variables are derived from the hematoxylin channel rather than the eosin channel of the cytoplasm. We confirmed from the original image that the signals of the hematoxylin channel of the cytoplasm were not from adjacent nuclei, as nuclear segmentation automatically shrinks the area of the cytoplasm if the two nuclei are close to each other. Eosin mainly stains the cytoplasm, and hematoxylin mainly stains the nuclei. The signals of hematoxylin were normally weak in the cytoplasm and can only be extracted using computer vision programs. Eosin is easily affected by the operation, and a slight change of dyeing time will cause the color to change. Although both hematoxylin and eosin channels were normalized, the features of the eosin channel were not so directly correlated with prognosis compared with the hematoxylin channels. This is slightly inconsistent with the previous finding of eosinophilic features. We think this is because both the eosinophilic and basophilic components increase in some high-grade ccRCC with decreased lipid components. While human naked eyes are more sensitive to eosin, pathologists label these ccRCCs with eosin.

## 5. Conclusion

In conclusion, we identified kurtosis in the hematoxylin channel of the cytoplasm as a positive prognostic predictor of ccRCC.

## Data Availability

The survival data generated or analyzed during this study are included within this article. The other data used to support the findings of this study are available from the corresponding author upon reasonable request.

## Supplementary Materials

Supplementary Materials Supplementary methods: DenseNet network structure, network hyperparameters, and training details.
