# Supplementary material for: Predicting Clear Cell Renal Cell Carcinoma Survival Using Kurtosis of Cytoplasm in the Hematoxylin Channel from Histology Slides
Source: J Oncol. 2022 Jan 13;2022:7693993. doi: 10.1155/2022/7693993 (PMC8776432; doi:10.1155/2022/7693993)
Supplement: Supplementary Materials — Supplementary methods: DenseNet network structure, network hyperparameters, and training details. [file 7693993.f1.docx]

**DenseNet Network Structure:**

__________________________________________________________________________________________________

Layer (type) Output Shape Param # Connected to

==================================================================================================

input_1 (InputLayer) (None, 112, 112, 3) 0

__________________________________________________________________________________________________

conv2d_1 (Conv2D) (None, 112, 112, 32) 864 input_1[0][0]

__________________________________________________________________________________________________

batch_normalization_2 (BatchNor (None, 112, 112, 32) 128 conv2d_1[0][0]

__________________________________________________________________________________________________

activation_2 (Activation) (None, 112, 112, 32) 0 batch_normalization_2[0][0]

__________________________________________________________________________________________________

conv2d_3 (Conv2D) (None, 112, 112, 16) 4608 activation_2[0][0]

__________________________________________________________________________________________________

concatenate_1 (Concatenate) (None, 112, 112, 48) 0 conv2d_1[0][0]

conv2d_3[0][0]

__________________________________________________________________________________________________

batch_normalization_3 (BatchNor (None, 112, 112, 48) 192 concatenate_1[0][0]

__________________________________________________________________________________________________

activation_3 (Activation) (None, 112, 112, 48) 0 batch_normalization_3[0][0]

__________________________________________________________________________________________________

conv2d_4 (Conv2D) (None, 112, 112, 16) 6912 activation_3[0][0]

__________________________________________________________________________________________________

concatenate_2 (Concatenate) (None, 112, 112, 64) 0 concatenate_1[0][0]

conv2d_4[0][0]

__________________________________________________________________________________________________

batch_normalization_4 (BatchNor (None, 112, 112, 64) 256 concatenate_2[0][0]

__________________________________________________________________________________________________

activation_4 (Activation) (None, 112, 112, 64) 0 batch_normalization_4[0][0]

__________________________________________________________________________________________________

conv2d_5 (Conv2D) (None, 112, 112, 16) 9216 activation_4[0][0]

__________________________________________________________________________________________________

concatenate_3 (Concatenate) (None, 112, 112, 80) 0 concatenate_2[0][0]

conv2d_5[0][0]

__________________________________________________________________________________________________

input_2 (InputLayer) (None, 200, 200, 3) 0

__________________________________________________________________________________________________

batch_normalization_5 (BatchNor (None, 112, 112, 80) 320 concatenate_3[0][0]

__________________________________________________________________________________________________

conv2d_2 (Conv2D) (None, 100, 100, 16) 2352 input_2[0][0]

__________________________________________________________________________________________________

activation_5 (Activation) (None, 112, 112, 80) 0 batch_normalization_5[0][0]

__________________________________________________________________________________________________

batch_normalization_1 (BatchNor (None, 100, 100, 16) 64 conv2d_2[0][0]

__________________________________________________________________________________________________

conv2d_6 (Conv2D) (None, 112, 112, 40) 3200 activation_5[0][0]

__________________________________________________________________________________________________

activation_1 (Activation) (None, 100, 100, 16) 0 batch_normalization_1[0][0]

__________________________________________________________________________________________________

average_pooling2d_1 (AveragePoo (None, 56, 56, 40) 0 conv2d_6[0][0]

__________________________________________________________________________________________________

max_pooling2d_1 (MaxPooling2D) (None, 50, 50, 16) 0 activation_1[0][0]

__________________________________________________________________________________________________

batch_normalization_6 (BatchNor (None, 56, 56, 40) 160 average_pooling2d_1[0][0]

__________________________________________________________________________________________________

batch_normalization_9 (BatchNor (None, 50, 50, 16) 64 max_pooling2d_1[0][0]

__________________________________________________________________________________________________

activation_6 (Activation) (None, 56, 56, 40) 0 batch_normalization_6[0][0]

__________________________________________________________________________________________________

activation_9 (Activation) (None, 50, 50, 16) 0 batch_normalization_9[0][0]

__________________________________________________________________________________________________

conv2d_7 (Conv2D) (None, 56, 56, 16) 5760 activation_6[0][0]

__________________________________________________________________________________________________

conv2d_10 (Conv2D) (None, 50, 50, 8) 1152 activation_9[0][0]

__________________________________________________________________________________________________

concatenate_4 (Concatenate) (None, 56, 56, 56) 0 average_pooling2d_1[0][0]

conv2d_7[0][0]

__________________________________________________________________________________________________

concatenate_6 (Concatenate) (None, 50, 50, 24) 0 max_pooling2d_1[0][0]

conv2d_10[0][0]

__________________________________________________________________________________________________

batch_normalization_7 (BatchNor (None, 56, 56, 56) 224 concatenate_4[0][0]

__________________________________________________________________________________________________

batch_normalization_10 (BatchNo (None, 50, 50, 24) 96 concatenate_6[0][0]

__________________________________________________________________________________________________

activation_7 (Activation) (None, 56, 56, 56) 0 batch_normalization_7[0][0]

__________________________________________________________________________________________________

activation_10 (Activation) (None, 50, 50, 24) 0 batch_normalization_10[0][0]

__________________________________________________________________________________________________

conv2d_8 (Conv2D) (None, 56, 56, 16) 8064 activation_7[0][0]

__________________________________________________________________________________________________

conv2d_11 (Conv2D) (None, 50, 50, 8) 1728 activation_10[0][0]

__________________________________________________________________________________________________

concatenate_5 (Concatenate) (None, 56, 56, 72) 0 concatenate_4[0][0]

conv2d_8[0][0]

__________________________________________________________________________________________________

concatenate_7 (Concatenate) (None, 50, 50, 32) 0 concatenate_6[0][0]

conv2d_11[0][0]

__________________________________________________________________________________________________

batch_normalization_8 (BatchNor (None, 56, 56, 72) 288 concatenate_5[0][0]

__________________________________________________________________________________________________

batch_normalization_11 (BatchNo (None, 50, 50, 32) 128 concatenate_7[0][0]

__________________________________________________________________________________________________

activation_8 (Activation) (None, 56, 56, 72) 0 batch_normalization_8[0][0]

__________________________________________________________________________________________________

activation_11 (Activation) (None, 50, 50, 32) 0 batch_normalization_11[0][0]

__________________________________________________________________________________________________

conv2d_9 (Conv2D) (None, 56, 56, 36) 2592 activation_8[0][0]

__________________________________________________________________________________________________

conv2d_12 (Conv2D) (None, 50, 50, 16) 512 activation_11[0][0]

__________________________________________________________________________________________________

average_pooling2d_2 (AveragePoo (None, 28, 28, 36) 0 conv2d_9[0][0]

__________________________________________________________________________________________________

average_pooling2d_3 (AveragePoo (None, 25, 25, 16) 0 conv2d_12[0][0]

__________________________________________________________________________________________________

batch_normalization_12 (BatchNo (None, 28, 28, 36) 144 average_pooling2d_2[0][0]

__________________________________________________________________________________________________

batch_normalization_15 (BatchNo (None, 25, 25, 16) 64 average_pooling2d_3[0][0]

__________________________________________________________________________________________________

activation_12 (Activation) (None, 28, 28, 36) 0 batch_normalization_12[0][0]

__________________________________________________________________________________________________

activation_15 (Activation) (None, 25, 25, 16) 0 batch_normalization_15[0][0]

__________________________________________________________________________________________________

conv2d_13 (Conv2D) (None, 28, 28, 16) 5184 activation_12[0][0]

__________________________________________________________________________________________________

conv2d_15 (Conv2D) (None, 25, 25, 8) 1152 activation_15[0][0]

__________________________________________________________________________________________________

concatenate_8 (Concatenate) (None, 28, 28, 52) 0 average_pooling2d_2[0][0]

conv2d_13[0][0]

__________________________________________________________________________________________________

concatenate_10 (Concatenate) (None, 25, 25, 24) 0 average_pooling2d_3[0][0]

conv2d_15[0][0]

__________________________________________________________________________________________________

batch_normalization_13 (BatchNo (None, 28, 28, 52) 208 concatenate_8[0][0]

__________________________________________________________________________________________________

batch_normalization_16 (BatchNo (None, 25, 25, 24) 96 concatenate_10[0][0]

__________________________________________________________________________________________________

activation_13 (Activation) (None, 28, 28, 52) 0 batch_normalization_13[0][0]

__________________________________________________________________________________________________

activation_16 (Activation) (None, 25, 25, 24) 0 batch_normalization_16[0][0]

__________________________________________________________________________________________________

conv2d_14 (Conv2D) (None, 28, 28, 16) 7488 activation_13[0][0]

__________________________________________________________________________________________________

conv2d_16 (Conv2D) (None, 25, 25, 8) 1728 activation_16[0][0]

__________________________________________________________________________________________________

concatenate_9 (Concatenate) (None, 28, 28, 68) 0 concatenate_8[0][0]

conv2d_14[0][0]

__________________________________________________________________________________________________

concatenate_11 (Concatenate) (None, 25, 25, 32) 0 concatenate_10[0][0]

conv2d_16[0][0]

__________________________________________________________________________________________________

batch_normalization_14 (BatchNo (None, 28, 28, 68) 272 concatenate_9[0][0]

__________________________________________________________________________________________________

batch_normalization_17 (BatchNo (None, 25, 25, 32) 128 concatenate_11[0][0]

__________________________________________________________________________________________________

activation_14 (Activation) (None, 28, 28, 68) 0 batch_normalization_14[0][0]

__________________________________________________________________________________________________

activation_17 (Activation) (None, 25, 25, 32) 0 batch_normalization_17[0][0]

__________________________________________________________________________________________________

global_average_pooling2d_1 (Glo (None, 68) 0 activation_14[0][0]

__________________________________________________________________________________________________

spatial_pyramid_pooling_1 (Spat (None, 672) 0 activation_17[0][0]

__________________________________________________________________________________________________

concatenate_12 (Concatenate) (None, 740) 0 global_average_pooling2d_1[0][0]

spatial_pyramid_pooling_1[0][0]

__________________________________________________________________________________________________

dense_1 (Dense) (None, 4) 2964 concatenate_12[0][0]

==================================================================================================

Total params: 68,308

Trainable params: 66,892

Non-trainable params: 1,416

**Network hyperparameters and training details**

nb_epoch = 100
img_rows, img_cols = 112, 112
img_channels = 3
dropout_rate = 0.0

depth=26
growth_rate=8
growth_rate2=4
bottleneck=False

reduction=0.5
dropout_rate=0.0
weight_decay=1e-4
subsample_initial_block=False
subsample_initial_block2=True

optimizer = Adam(lr=0.5e-4)

loss='categorical_crossentropy'

optimizer.metrics="accuracy"
